# LSTM‐Based Recurrent Neural Network Predicts Influenza‐Like‐Illness in Variable Climate Zones

**DOI:** 10.1002/iid3.70367

**Published:** 2026-02-23

**Authors:** Alfred Amendolara, Christopher Gowans, Joshua Barton, Andrew Payne, David Sant

**Affiliations:** ^1^ Department of Biomedical Science Noorda College of Osteopathic Medicine Provo Utah USA

**Keywords:** influenza, LSTM, modeling, neural network

## Abstract

**Background:**

Influenza virus is responsible for a recurrent, yearly epidemic in most temperate regions of the world. Flu has been responsible for a high disease burden in recent years, despite the confounding presence of SARS‐CoV‐2. However, the mechanisms behind seasonal variance in flu burden are not well understood. This study seeks to expand understanding of the impact of variable climate regions on seasonal flu trends. To that end, three climate regions have been selected. Each region represents a different ecological zone and provides different weather patterns.

**Methods:**

A long short‐term memory (LSTM)‐based recurrent neural network was used to predict influenza‐like‐illness trends for three separate locations: Hawaii, Vermont, and Nevada. Flu data were gathered from the Center for Disease Control as weekly influenza‐like‐illness (ILI) percentages. Weather data were collected from Visual Crossing and included temperature, wind speed, UV index, solar radiation, precipitation, and humidity. Data were prepared and the model was trained as described previously.

**Results:**

All three regions showed strong seasonality of flu trends with Hawaii having the largest absolute ILI values. Temperature showed a moderate negative correlation with ILI in all three regions (Vermont = −0.54, Nevada = −0.56, Hawaii = −0.44). Humidity was moderately correlated in Nevada (0.47) and weakly correlated with ILI in Hawaii (0.22). Vermont ILI did not correlate with humidity. Precipitation and wind speed were weakly correlated in all three regions. Solar radiation and UV index showed moderate correlation in Vermont (−0.33, −0.36) and Nevada (−0.53, −0.55), but only a weak correlation in Hawaii (−0.15, −0.18). When trained on the complete data sets, baseline model performances for all three datasets at +1 week were equivalent. Models trained on one region and used to predict cross‐regional data performed uniformly and equivalent to baseline.

**Conclusions:**

Results indicate that climate variables were weak to moderate predictors in all regions. Initial modeling attempts revealed uniform performance in all regions. Despite strong climate differences, cross‐regional LSTM models performed comparably, suggesting that seasonal patterns, rather than absolute climate variables, drive ILI trends. Additionally, this data suggests that absolute climate changes may not be influential as relative seasonal changes.

**Abbreviations**
CDC: Center for Disease Control and Prevention
COVID‐19: coronavirus disease 2019
ILI: influenza‐like‐illness
LSTM: long short‐term memory
MSE: mean squared error

## 1 Introduction

Influenza‐like illness (ILI) is a major contributor to morbidity and mortality each year and represents an ongoing challenge to public health policy and infrastructure [1]. With the recent coronavirus disease 19 (COVID‐19) pandemic, the burden and influence of the flu has become more complex. Recent research has shown COVID‐19 and ILI share epidemiological features, such as seasonality cycles [2]. While COVID‐19 has tapered since its initial appearance, it is here to stay, and the flu has returned to pre‐pandemic incidence levels. Despite the temporaril decreased incidence during pandemic lockdowns, flu will likely continue to cause significant disease burden. Understanding the factors that drive seasonal influenza transmission is essential for improving prediction models and informing proactive public health interventions.

A number of environmental variables, including temperature, humidity, UV index, and solar radiation, correlate with seasonal variation in ILI incidence [2]. Geographic differences in climate, such as those between tropical, temperate, and arid zones, have been shown to produce distinct patterns in influenza prevalence and peak timing [3]. This suggests that climate‐specific models may be required to accurately forecast regional flu trends. To investigate this, we selected three U.S. states representing distinct, homogenous climate archetypes: Hawaii, Vermont, and Nevada. Hawaii provides uniform tropical data sets, Vermont contributes a temperate four‐season climate, and Nevada, being 75% arid, provides a desert climate. While more climates are available in the United States and elsewhere, our current investigation was limited to these three states to provide a simplified scaffolding to test our hypothesis by reducing variability in climate patterns and maintaining consistency in data availability and quality. By focusing on these regions, we aimed to explore the influence of local climate variability on influenza trends while testing the generalizability of our predictive model trained in one setting and applied to another.

Beyond climate variables, many other factors contribute to the spread of influenza, including human travel patterns and air pollution [2, 3, 4, 5, 6]. This presents challenges when developing tools to predict flu trends, an important consideration when managing the sizable disease burden the flu causes each year [7].

Artificial neural networks are effective architectures for disease modeling and provide the ability to capture non‐linear relationships related to a number of infectious diseases [8, 9, 10]. Recurrent neural networks (RNNs), and specifically long short‐term memory (LSTM) architectures, have become increasingly prominent tools for forecasting influenza [11]. LSTM nodes seek to solve the disappearing or exploding gradient issue found commonly with neural networks, especially when dealing with highly dimensional time series data, through the inclusion of a constant error carousel as well as a “forget gate.” Both features allow the LSTM node to reset occasionally, while still retaining its time‐dependent memory [12, 13]. They are particularly adept at capturing patterns in time‐dependent sequential data such as stock price trends, weather, and disease incidence. This strength lends itself to disease forecasting, especially when considering covariate weather data. A number of flu modeling systems in recent years have used LSTM, on its own or in combination with other architectures, to successfully predict flu patterns [11, 14, 15, 16, 17, 18].

Building upon our previous work, we applied an LSTM‐based recurrent neural network to predict ILI rates across three distinct climate regions in the U.S. Our primary goal was to determine how regional climate variables influence the performance of these models, and whether models trained in one climate zone retain predictive utility when applied cross‐regionally. This approach allows for evaluation of not only the impact of specific climate variables on flu transmission but also the broader question of whether climate plays a causal role or merely acts as a proxy for other seasonal factors.

We hypothesized that models trained on flu and climate data from one region would perform poorly when applied to another with a distinct climate profile, due to disparate seasonal variation. By testing this hypothesis, our study aims to provide new insights into the portability of time‐series modeling approaches for influenza and to clarify the relative importance of climate in shaping flu dynamics.

## 2 Materials and Methods

These models were designed and constructed in Python v3.9.13 using TensorFlow v2.10 and the Keras API running natively on Windows 11. The computer used to train and run the model has the following specifications: AMD Ryzen 9 5900X @ 3.70 GHz, 64GB 3600 MHz DDR4 RAM, Nvidia RTX 3060 Ti 8GB.

### 2.1 Data Collection and Preparation

Flu data were gathered from the CDC as weekly unweighted ILI percent for Vermont, Nevada, and Hawaii [1]. Data was downloaded in CSV format. Data spanned from week 40 of 2010 to week 34 of 2023. Weather data were acquired over the same time period from Visual Crossing and included temperature, wind speed, UV index, solar radiation, precipitation, and humidity [19]. These weather data sets were chosen based on previous reports, including our findings presented in Amendolara et al. 2023.

Data were prepared using Excel and Python. Once data was cleaned and compiled, it was normalized using z‐score normalization (Equation 1), a time series with a lag of 4 weeks was created, and it was reshaped into a 3‐dimensional array for use with the model. Data was normalized independently for each training and testing set to reduce data leakage. This time lag was chosen as it outperformed time lags of ‐1, ‐12, ‐16, and ‐52 weeks in earlier validation testing [20].

(1) $$\frac{\left(x-\mathrm{mean}\right)}{\mathrm{standard}\;\mathrm{deviation}}$$

**Figure 1:**
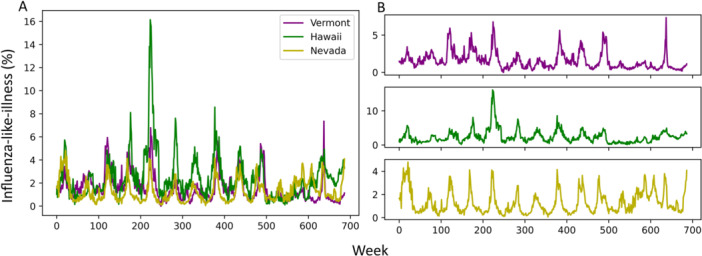
Vermont, Nevada, and Hawaii show similar seasonal flu trends. (A) Overlayed data from all three states. (B) Separated data per state. Top: Vermont, Middle: Hawaii, Bottom: Nevada.

Flu data for each region were visualized (Figure 1). Additionally, climate data was visualized (Supporting Information Figures 1–3) and a correlation matrix was generated to assess the relationship between flu data and climate variables (Table 1).

**Table 1:** Correlation matrices.

Vermont									
	% ILI	Temp max	Temp min	Temp	Humidity	Precipitation	Wind speed mean	Solar radiation	UV index
% ILI	1.00								
Temp max	−0.53	1.00							
Temp min	−0.54	0.97	1.00						
Temp	−0.54	0.99	0.99	1.00					
Humidity	−0.01	0.04	0.16	0.09	1.00				
Precipitation	−0.13	0.23	0.29	0.26	0.37	1.00			
Wind speed mean	0.19	−0.42	−0.41	−0.41	−0.41	−0.04	1.00		
Solar radiation	−0.34	0.79	0.71	0.77	−0.28	0.09	−0.23	1.00	
UV index	−0.36	0.81	0.73	0.78	−0.30	0.08	−0.22	0.97	1.00

Nevada									
	% ILI	Temp max	Temp min	Temp	Humidity	Precipitation	Wind speed mean	Solar radiation	UV index
% ILI	1.00								
Temp max	−0.56	1.00							
Temp min	−0.55	0.92	1.00						
Temp	−0.57	0.98	0.97	1.00					
Humidity	0.47	−0.84	−0.69	−0.79	1.00				
Precipitation	0.11	−0.32	−0.16	−0.25	0.41	1.00			
Wind speed mean	−0.09	−0.05	0.15	0.07	−0.10	0.30	1.00		
Solar radiation	−0.53	0.68	0.68	0.71	−0.67	−0.23	0.19	1.00	
UV index	−0.55	0.75	0.71	0.76	−0.74	−0.29	0.20	0.90	1

Hawaii									
	% ILI	Temp max	Temp min	Temp	Humidity	Precipitation	Wind speed mean	Solar radiation	UV index
% ILI	1.00								
Temp max	−0.43	1.00							
Temp min	−0.45	0.91	1.00						
Temp	−0.45	0.98	0.97	1.00					
Humidity	0.22	−0.41	−0.32	−0.34	1.00				
Precipitation	0.03	−0.24	−0.12	−0.17	0.48	1.00			
Wind speed mean	−0.24	0.29	0.45	0.33	−0.60	−0.19	1.00		
Solar radiation	−0.15	0.36	0.26	0.31	−0.38	−0.27	0.18	1.00	
UV index	−0.18	0.44	0.29	0.37	−0.46	−0.36	0.17	0.91	1.00

### 2.2 Model

Three identical models were constructed for this paper designed to predict next ( + 1) week ILI values. The general model architecture has been previously described and validated. Hyperparameters were tuned via manual testing and the most performant values were used. Additionally, this model architecture outperformed multiple linear regression, k‐nearest neighbor, gradient boosting, extreme gradient boosting, and multi‐layer perceptron models on +1‐week predictions when predicting flu data from New England [20, 21].

In brief, the model was built with a variable input‐shape, bidirectional 500 node input layer, two bidirectional 500 node LSTM hidden layers and a variable shaped dense output layer. This model architecture was built iteratively, starting with minimum viable layer number and size and optimizing for performance. Select model parameters are presented below:

– Max Epochs (early stopping implemented): 500
– Batch Size: 270
– Validation Split: 0.2
– Initial Learning Rate: 0.001
– Minimum Learning Rate: 0.000000001
– Optimizer: Adam

Three instances of the model were trained. The first 600 weeks of each regional data (2010 week 40 – 2022 week 12) set were used for training and 74 weeks (2022 week 13 – 2023 week 34) were reserved for final testing. Each model was trained on 80% of their respective training data with 20% reserved for training validation. Baseline model performance in each region was evaluated by calculating per‐prediction error as well as mean squared error (MSE) for each same‐region test set. A Kruskal‐Wallis H‐test was performed to ensure that error was not significantly different at baseline between the three models. This test was chosen given that the residuals were non‐normally distributed and of unequal variance based on Shapiro‐Wilke and Levene tests, respectively. All models were validated using a k‐fold cross validation with *k* = 10.

In order to assess generalizability and differences in regional climate influence, trained models were used to predict 400–600 weeks of data from each of the other two regions i.e., the model trained on Vermont data was used to predict data from Hawaii and data from Nevada separately. Overall model performance was evaluated using MSE and compared. Hawaii and Vermont test sets ranged from 2010 week 40 – 2022 week 12. Nevada included a slightly shorter test set to avoid influence of notable pandemic‐era ILI variability and ranged from 2010 week 40 – 2018 week 21.1

All data and code used in this report are available via GitHub and Zenodo: https://zenodo.org/doi/10.5281/zenodo.13294740.

## 3 Results

### 3.1 Seasonal Flu Trends Are Similar Throughout Regions

ILI patterns were largely similar between regions. Hawaii had one unusually high amplitude spike starting at approximately week 200, but the data from all three regions otherwise showed similar seasonality (Figure 1). COVID‐19 pandemic restrictions are likely reflected started around the 500‐week mark, where a departure from the regular seasonal pattern may be observed in all three regions. Weather data followed a similar pattern. Hawaii displayed less overall amplitude change in temperature, solar radiation, and UV index across the year. Absolute values of climate variables differed as well. However, all three regions still displayed underlying seasonal patterns. Plotted data from each region may be viewed in Additional File 1.

### 3.2 Climate Variables Correlate with IlI Differently in Each Region

Temperature showed a moderate negative correlation with ILI in all three regions (Vermont = −0.54, Nevada = −0.56, Hawaii = −0.44). Humidity was moderately correlated in Nevada (0.47) and weakly correlated with ILI in Hawaii (0.22). Vermont ILI did not correlate with humidity. Precipitation and wind speed were weakly correlated in all three regions. Solar radiation and UV index showed moderate correlation in Vermont (−0.34, −0.36) and Nevada (−0.53, −0.55), but only weak correlation in Hawaii (−0.15, −0.18) (Table 1).

### 3.3 Baseline Performance Is Similar Across Models

**Figure 2:**
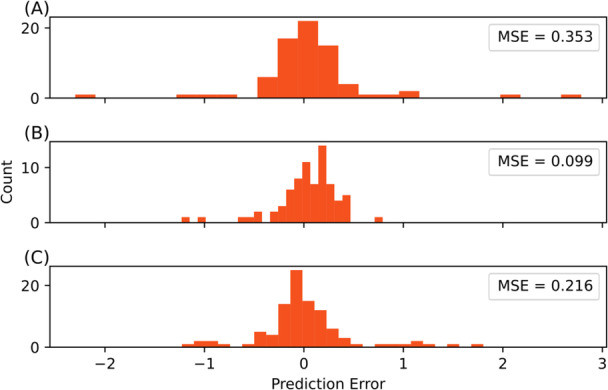
Baseline performance on same‐region test data is Variable but Statistically Similar. (A) Vermont‐trained model predicting Vermont test data. (B) Hawaii‐trained model predicting Hawaii test data. (C) Nevada‐trained model predicting Nevada test data. Kruskal–Wallis H‐test *p‐*value = 0.0794. MSE = mean squared error.

When predicting ILI rates on same‐region test data, all three models performed similarly at baseline. The Vermont model was able to achieve a MSE of 0.353, Hawaii a MSE of 0.099, and Nevada a MSE of 0.216. Error distribution looks broadly uniform between models and a Kruskal–Wallis H‐test shows that they are not statistically different (*p‐*value = 0.0794) (Figure 2).

### 3.4 Models Predict Cross‐Regional Data Equally Well

Testing on the larger, cross‐region data set produced in general similar MSE compared to baseline performance. Hawaii‐ and Nevada‐trained models did experience slightly decreased performance, though still within a reasonable range compared to baseline (Table 2).

**Table 2:** Comparison of cross‐regional performance. Presented as mean squared error at +1‐week prediction.

	Test set (*n* = 600)			
		Vermont	Hawaii	Nevada
Training set	Vermont	—	0.179	0.239
	Hawaii	0.254	—	0.296
	Nevada	0.258	0.244	—

**Figure 3:**
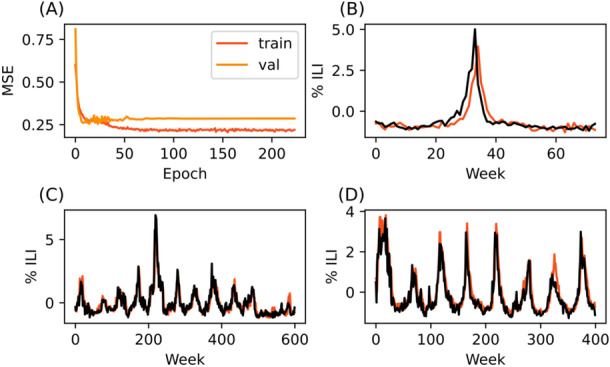
LSTM model trained on data from Vermont is able to predict trends in Hawaii and Nevada. (A) Training and validation curve. (B) 1+ week predictions made on seventy‐four weeks of reserved Vermont training data (C) 1+ week predictions made on Hawaii data (D) 1+ week predictions made on Nevada data. Black lines represent CDC‐reported data, while red lines indicate model predictions.

The best prediction performance was achieved by the Vermont‐trained model predicting Hawaii data (MSE 0.179). This was also a significant performance improvement from the Vermont baseline performance (Baseline MSE = 0.353). In fact, both cross‐regional predictions improved the Vermont model's MSE (Table 2, Figure 3).

**Figure 4:**
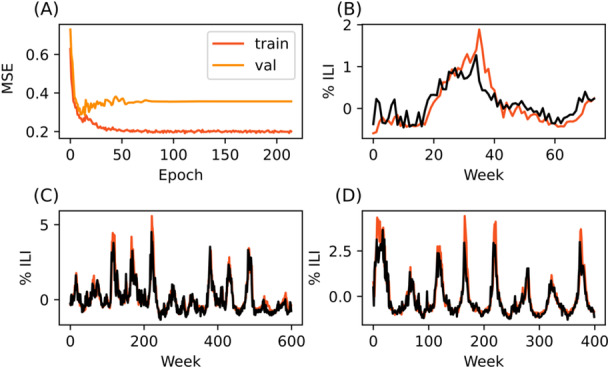
LSTM model trained on data from Hawaii is able to predict trends in Vermont and Nevada. (A) Training and validation curve. (B) 1+ week predictions made on seventy‐four weeks of reserved Hawaii training data (C) 1+ week predictions made on Vermont data (D) 1+ week predictions made on Nevada data. Black lines represent CDC‐reported data, while red lines indicate model predictions.

Nevada‐trained models performed similarly to baseline on both test sets, and performance was generally unremarkable. Visually though, the Nevada‐trained model appears to provide the most consistent predictions with little overshooting or undershooting of true values (Figure 4).

**Figure 5:**
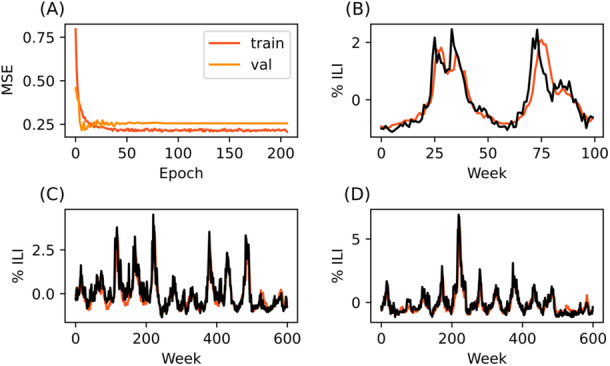
LSTM model trained on data from Nevada is able to predict trends in Hawaii and Nevada. (A) Training and validation curve. (B) 1+ week predictions made on approximately one hundred weeks of reserved Nevada training data (C) 1+ week predictions made on Vermont data (D) 1+ week predictions made on Hawaii data. Black lines represent CDC‐reported data, while red lines indicate model predictions.

The worst prediction performance occurred when using the Hawaii‐trained model to predict Nevada data (Table 2, Figure 5). However, this performance was still comparable to baseline performance. It is likely these performance differences are not meaningful.

Despite the slight variation between model performance, overall, each model performed very similarly and well within the spread of baseline performance.

## 4 Discussion

This study aimed to evaluate the predictive power and generalizability of LSTM‐based neural networks for modeling ILI across regions with distinct climates. We found that models trained in one region (e.g., Vermont) could predict flu trends in other regions (e.g., Hawaii and Nevada) with comparable performance to their base test sets. This uniformity in cross‐regional performance was observed despite substantial differences in absolute temperature, humidity, solar radiation, and other climate variables. This suggests that these environmental factors may not be the primary drivers of seasonal flu trends.

Previously, we demonstrated through ablation analysis that climate variables did improve model performance, which, given our model architecture is to be expected. However, when the model was trained on synthetic data with modified seasonality, predictions break down significantly [20]. Combined with our current results, this suggests that seasonal structure, not absolute climate values, governs ILI trends. Interestingly, ILI data are very similar between all three regions. This is somewhat counterintuitive, as previous research has shown that tropical climates tend to have flatter flu patterns and less seasonal variation [22]. Additionally, while climate variables such as temperature and humidity have been previously linked to flu seasonality, as has climate region [2, 3], little performance difference between our models was observed. The convergence of model performance across climates raises the possibility that temporal factors such as behavioral changes, school schedules, or synchronized travel patterns may be more influential than absolute climate values. Hawaii's flu incidence, for instance, may be heavily impacted by travel as well as its relatively small population [23].

All three models performed similarly at baseline. There is some variability in MSE and error distribution, but this is to be expected as all three models were validated on slightly different test sets. This suggests that the model is robust to variations in training data regarding climate factors, and that the magnitude of change in climate variables is less important to model performance than the pattern of the change. For example, although Vermont experiences a 76 °F annual temperature swing compared to just 15 °F in Hawaii, both regions yielded similar predictive outcomes. This aligns with our previous findings that altering the phase of seasonal inputs, rather than their values, has a significant impact on model performance [20]. It may be that the strong seasonal shifts help encode these patterns in the model. And thus, we see strong performance when cross‐testing even in regions without traditional seasons.

The results presented here position climate variables as correlated indicators of flu trends rather than mechanistic drivers. This distinction is important, particularly for understanding whether climate itself causes ILI fluctuations. Notably, the strongest climate‐ILI correlations were observed in Vermont and Nevada, which have more pronounced seasons. In contrast, Hawaii showed weaker associations despite comparable seasonal ILI values. This raises the question of whether flu follows the seasons due to environmental pressures or due to synchronized patterns in human activity. On a biological level, there is evidence in lab environments that climate variables do impact flu transmission and spread [20]. But there exists a strong possibility that, due to the complicated nature of disease transmission and modern travel patterns, climate is less relevant in modern populations. That is to say that population‐based factors are significantly more important than climate factors when considering yearly flu spread and burden. Notably, a recent paper showed that flu incidence in Puerto Rico had synced with the mainland United States, lending credence to this hypothesis [24]. This is further supported by the trends seen in the recent COVID‐19 pandemic. As is apparent in the ILI data presented here, flu burden dropped notably in the face of pandemic restrictions and precautions.

There are therefore practical implications for influenza modeling. Our findings suggest that time‐series models trained in data‐rich, high‐surveillance areas can be applied to other regions, even with different climate conditions, without substantial loss in performance. This could be valuable in settings with limited public health infrastructure or inconsistent flu reporting. Additionally, these results support the idea that predictive accuracy can be achieved without finely tuning models to absolute climate values, allowing for more generalized models that are easier to maintain and deploy.

So, climate variables may serve as a predictor rather than a driver. The implications of this are somewhat difficult to interpret. Practically, it may not matter since they serve as a valuable data source for predictive models. However, it does indicate that utilizing climate data for predictive modeling may be less useful in the long run that developing network‐based models incorporating population level data.

Including population‐based data may improve performance past what is achievable with just climate and disease data. Topirceanu and Precup developed a model using geo‐hierarchical population mobility to model recurrent disease spread [25]. Additionally, accounting for school schedule variability and holidays may provide increased predictive power [26]. The best model performance would likely be achieved by a model combining spatially‐aware population data, vaccine coverage rates, and climate factors.

### 4.1 Limitations

This report does suffer from several limitations. Notably, Covid‐19 is now present and practically impossible separate from flu in ILI datasets. As this is expected to continue in future years, we avoided attempts to systematically correct for its influence. However, the presence of Covid‐19, and especially its effect on flu patterns during the pandemic, may impact the conclusions presented here. Reassuringly, our model proved robust to the variation in normal seasonal patterns during those pandemic years in Vermont and Hawaii, though Nevada's data was truncated somewhat to account for variability likely due to increased pandemic influence when testing cross‐trained models.

Data were also limited to weekly flu and weather data that were available in an overlapping time period as well as recorded at the same time scale. Including more than the currently available 12 years may improve performance, though perhaps not to a significant degree. This is not a major limitation but may be a consideration when evaluating model performance. Additionally, we choose not to normalize data across states to avoid data leakage during training, however variable ILI amplitudes present may itself introduce a level of bias.

Aside from limited data, which is an ongoing challenge when combining data from multiple separate sources, the regions (in this case individual states) are not perfect proxies for their climate zones. ILI rates are recorded statewide. Climate data, on the other hand, is often aggregated from one or a handful of weather stations. This creates inherent noise and imprecision in the data. Furthermore, states are not uniform and may have variations in climate and weather patterns depending on location, elevation, and other environmental factors. This is a limitation of any model using data on this scale and is largely unavoidable. It does likely explain some of the minor inconsistencies in performance as well as the weaker correlation between certain variables and ILI.

Finally, this report does not consider other potential variables that may impact flu spread. It appears, based on the results presented and existing literature, that there are other significant factors that drive flu trends including pollen, travel, indoor environmental variable, and other concurrent diseases [2, 3, 4, 5, 6]. Additional work, likely with a completely different modeling approach will be required to delineate the impact of population‐level factors.

### 4.2 Future Applications

The results of this paper may be applied or extended in several ways. Practically, we have shown that this modeling approach is robust to variations in climate and flu data. This may allow a model that has been trained on data from a region with high quality, consistent flu reporting and reliable weather stations to be applied to a region without those resources. Thus, predictions could be made, and potential flu burden assessed, without the need to make a bespoke model. This could be especially useful in areas where flu data is limited or has only recently begun being tracked.

In addition, these findings may help refocus modeling efforts toward integrating population‐level and behavioral data, which may prove to be more fundamental in driving flu dynamics. Future work should explore how incorporating variables such as school calendars, mobility data, or social distancing patterns could improve both the accuracy and interpretability of predictive models.

This, or similar models, could also be extended into a network‐based approach where multiple, linked models trained on data from disparate cities or states inform the ILI predictions for a region of interest.

Finally, this model has been validated and is performant enough to consider optimization and deployment for real world applications. We did not formally collect runtime or computational load data during these experiments; however, model training time averaged less than 2 min, and individual predictions took several milliseconds. This model, or a model like this, could be deployed with minimal refactoring for near real time prediction on intermediate hardware similar to our reported system.

## 5 Conclusions

Given the known limitations of the modeling approach presented here, it is difficult to draw strong conclusions about the underlying epidemiological processes of flu trends. However, our results may provide several insights for both practical modeling and theoretical understanding of flu patterns. First, we demonstrate that LSTM‐based models are robust to data variation and highly generalizable across climate regions. This supports the utility of such models for short‐term ILI forecasting, even when trained on data from a different geographic or climatic context. Second, while climate variables such as temperature, humidity, and solar radiation were moderately correlated with ILI in some regions, their predictive power did not appear to stem from their absolute values. Instead, our results suggest that the seasonal pattern of these variables plays a more influential role in forecasting ILI trends. Further research is needed to delineate the impact of climate versus other population variables. This is particularly important when deciding on public health policy, predicting flu burden, and developing strategies to reduce said burden.

## Author Contributions

**Alfred Amendolara:** conceptualization, investigation, writing – original draft; methodology, visualization, writing – review and editing, formal analysis, data curation. **Christopher Gowans:** data curation. **Joshua Barton:** data curation. **Andrew Payne:** writing – original draft, writing – review and editing, supervision. **David Sant:** writing – original draft, writing – review and editing, validation, supervision.

## Funding

The authors received no specific funding for this work.

## Ethics Statement

The authors have nothing to report.

## Consent

The authors have nothing to report.

## Conflicts of Interest

The authors declare no conflicts of interest.

## Supporting information


**Supporting Figure 1:** Hawaii Climate Data. **Supporting Figure 2:** Nevada Climate Data. **Supporting Figure 3:** Vermont Climate Data.

## Data Availability

The data that support the findings of this study are openly available in Zenodo at https://doi.org/10.5281/zenodo.13294741. All data and code described in this paper are available via GitHub or Zenodo at https://zenodo.org/doi/10.5281/zenodo.13294740.
