# Supplementary material for: LSTM‐Based Recurrent Neural Network Predicts Influenza‐Like‐Illness in Variable Climate Zones
Source: Immun Inflamm Dis. 2026 Feb 23;14(2):e70367. doi: 10.1002/iid3.70367 (PMC12928072; doi:10.1002/iid3.70367)
Supplement: Supplementary file 1 — Supporting Figure 1: Hawaii Climate Data. Supporting Figure 2: Nevada Climate Data. Supporting Figure 3: Vermont Climate Data. [file IID3-14-e70367-s001.docx]

Supplemental Materials


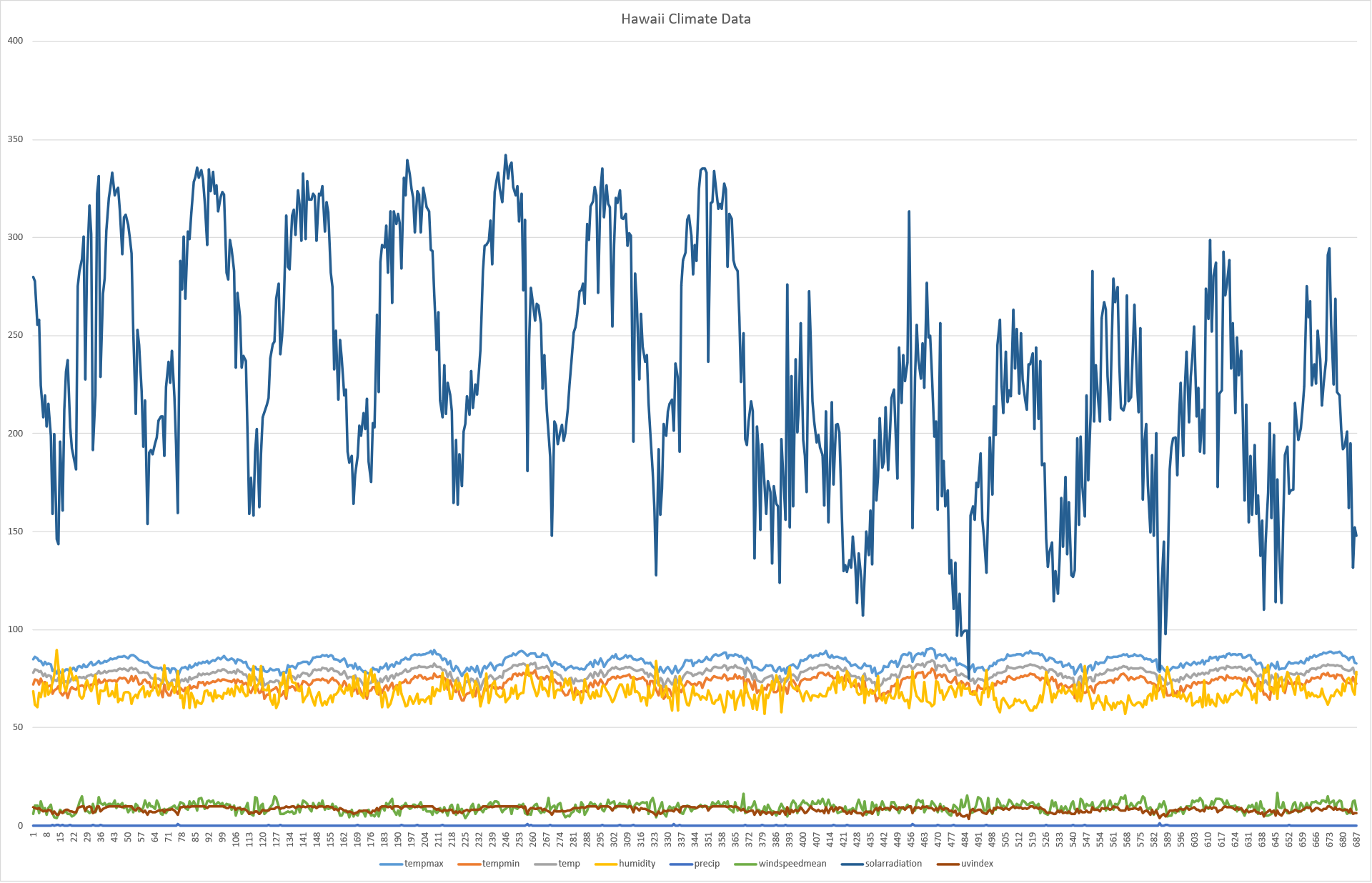


Supplemental Figure 1. Hawaii Climate Data


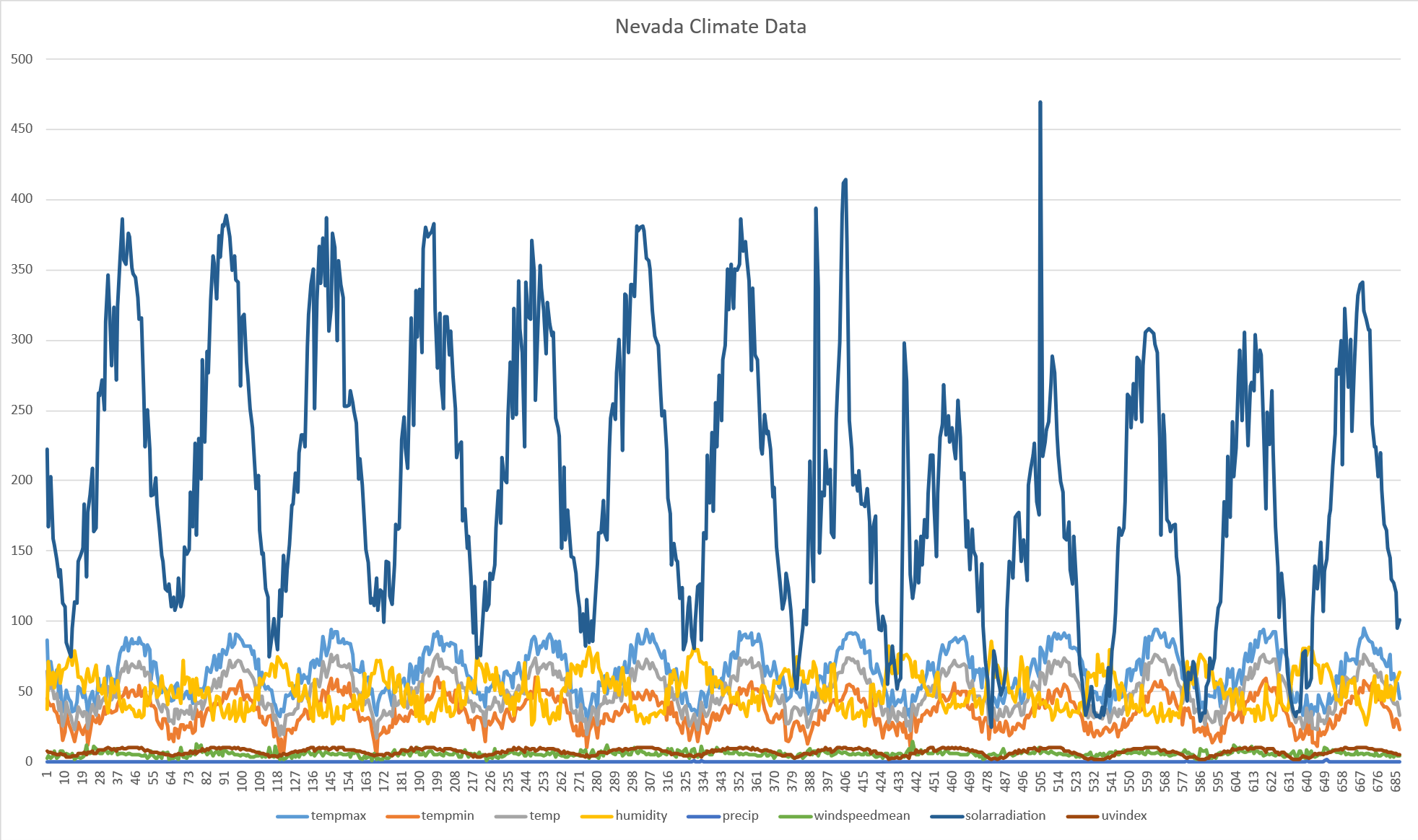


Supplemental Figure 2. Nevada Climate Data


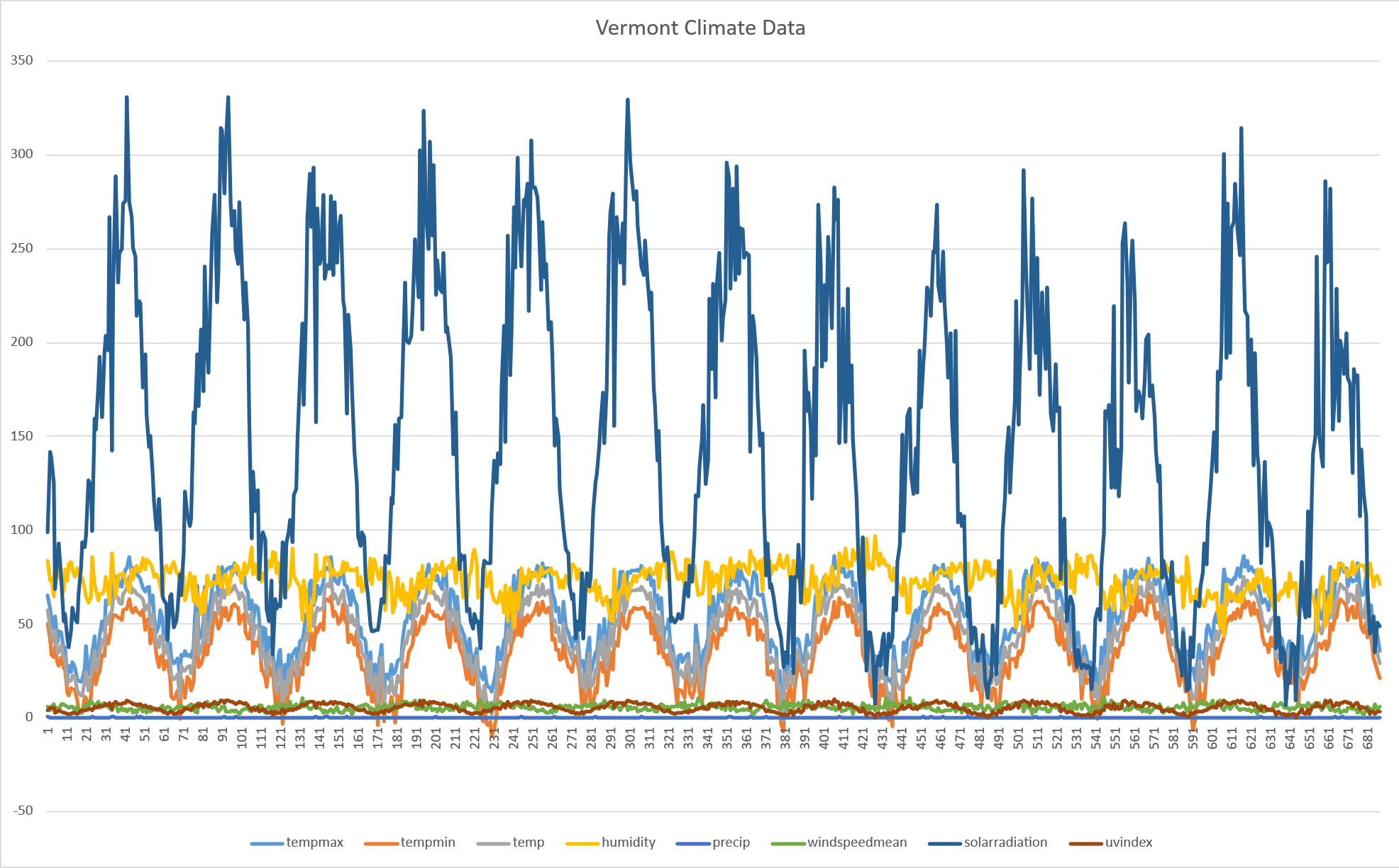


Supplemental Figure 3. Vermont Climate Data
